# A Handbook of Therapeutics

**Published:** 1898-09

**Authors:** 


**A Handbook of Therapeutics.** By SYDNEY RINGER, M.D., F.R.S., and HARRINGTON SAINSBURY, M.D. Thirteenth Edition. Pp. xi., 746. London: H. K. Lewis. 1897.

A book so well known as Ringer's *Therapeutics* needs no commendation; it has been a standard work of reference for many years. The present edition has been extensively revised, and several entirely fresh sections have been added, such as those on serum therapeutics and invalid dietary. An examination of many of the chapters on individual drugs or groups of remedies assures us that the work has been carefully brought up to recent date, and it deserves to continue to represent the typical English text-book of therapeutics. Dr. Ringer, as is well known, relies much more upon clinical observation than experimental research as a guide to the action and uses of remedies, but he and Dr. Sainsbury by no means despise investigation by physiological method. Every intelligent practitioner should possess a copy of this storehouse of information and suggestion as to treatment.

